# MRI subtypes in Parkinson’s disease across diverse populations and clustering approaches

**DOI:** 10.1038/s41531-024-00759-2

**Published:** 2024-08-16

**Authors:** Anna Inguanzo, Rosaleena Mohanty, Konstantinos Poulakis, Daniel Ferreira, Barbara Segura, Franziska Albrecht, J-Sebastian Muehlboeck, Tobias Granberg, Henrik Sjöström, Per Svenningsson, Erika Franzén, Carme Junqué, Eric Westman

**Affiliations:** 1 Division of Clinical Geriatrics, Center for Alzheimer Research, Department of Neurobiology, Care Sciences, and Society, Karolinska Institutet, Stockholm, Sweden; 2 Medical Psychology Unit, Department of Medicine, Institute of Neurosciences, University of Barcelona, Barcelona, Catalonia Spain; 3 Facultad de Ciencias de la Salud. Universidad Fernando Pessoa Canarias, Las Palmas, Spain; 4 Fundació de Recerca Clínic Barcelona, Institut d’Investigacions Biomèdiques August Pi i Sunyer (FRCB-IDIBAPS), Barcelona, Catalonia Spain; 5 Centro de Investigación Biomédica en Red sobre Enfermedades Neurodegenerativas, Barcelona, Catalonia Spain; 6 Division of Physiotherapy, Department of Neurobiology, Care Sciences and Society, Karolinska Institutet, Stockholm, Sweden; 7 Karolinska University Hospital, Women’s Health and Allied Health Professionals Theme, Medical unit Occupational Therapy & Physiotherapy, Stockholm, Sweden; 8 Division of Neuro, Department of Clinical Neuroscience, Karolinska Institutet, Stockholm, Sweden; 9 Department of Neuroradiology, Karolinska University Hospital, Stockholm, Sweden; 10 Center for Neurology, Academic Specialist Center, Stockholm, Sweden; 11 Basic and Clinical Neuroscience, Institute of Psychiatry, Psychology and Neuroscience, Kings College London, London, UK; 12 Department of Neuroimaging, Center for Neuroimaging Sciences, Institute of Psychiatry, Psychology and Neuroscience, Kings College London, London, UK

**Keywords:** Parkinson's disease, Diagnostic markers

## Abstract

Parkinson’s disease (PD) is clinically heterogeneous, which suggests the existence of subtypes; however, there has been no consensus regarding their characteristics. This study included 633 PD individuals across distinct cohorts: unmedicated de novo PD, medicated PD, mild-moderate PD, and a cohort based on diagnostic work-up in clinical practice. Additionally, 233 controls were included. Clustering based on cortical and subcortical gray matter measures was conducted with and without adjusting for global atrophy in the entire PD sample and validated within each cohort. Subtypes were characterized using baseline and longitudinal demographic and clinical data. Unadjusted results identified three clusters showing a gradient of neurodegeneration and symptom severity across the entire sample and the individual cohorts. When adjusting for global atrophy eight clusters were identified in the entire sample, lacking consistency in individual cohorts. This study identified atrophy-based subtypes in PD, emphasizing the significant impact of global atrophy on subtype number, patterns, and interpretation in cross-sectional analyses.

## Introduction

Parkinson’s disease (PD) is a neurodegenerative disorder characterized by a wide variety of motor and non-motor symptoms, including cognitive impairment^1^. Given the heterogeneous nature of PD, the identification of subtypes is crucial. In this context, unsupervised cluster analysis has emerged as a promising approach to identify subtypes in neurodegenerative disorders^2^. Early PD studies relied on clinical, demographic and/or cognitive variables to define subtypes^3,4^; however, to determine whether these subtypes manifest variations in disease mechanisms it is fundamental to incorporate objective biological measures^5^. Along these lines, a few key studies have used structural magnetic resonance imaging (sMRI) to identify PD subtypes with distinct patterns of brain atrophy^6–10^.

However, the existing literature primarily consists of small-scale studies and lacks consensus on the specific number, as well as the biological and clinical characteristics of the subtypes. Clinically, PD can progress from de novo stage (unmedicated with little/mild symptoms) to a more severe stage requiring dopaminergic medication for symptom management. Some studies have identified three atrophy subtypes^6,8^, whereas other studies have identified two^7,11^. These studies have focused on either de novo patients or patients in relatively advanced stages undergoing treatment for PD, but not both simultaneously. Further, there are methodological differences in how the studies identify subtypes. Previous studies differed in the clustering method and the input data used for clustering, as some exclusively focused on atrophy measures from cortical brain regions^6,7^, while other studies included both cortical and subcortical data^8,10^. Further, some studies did consider variability in global atrophy and adjusted for it^6^, while others did not^8^. While there is no widely accepted standard for whether or not to adjust for global atrophy, the implications of such adjustment for subtyping remain unclear due to a lack of a head-to-head comparison. To identify robust biological subtypes of any disease, it is important to be inclusive of a broader spectrum of the disease course, which has been lacking in PD. Such inclusivity, however, raises the challenge of effectively disentangling whether biological subtypes capture distinct disease-specific patterns or merely different stages of the disease. To this end, Ferreira et al.^12^ proposed a framework conceptualizing biological subtypes of Alzheimer’s disease (AD) with two key dimensions—*typicality*, capturing the deviation of observed atrophy from the expected typical atrophy pattern in the disease and *severity* as a biological measure capturing overall neurodegeneration or disease progression. Given the neurodegenerative nature common to both diseases, whether such a framework may extend to PD remains to be validated.

Therefore, the goal of this study was to implement head-to-head robust and reproducible methods to capture sMRI subtypes that can be applied on samples encompassing a broader PD spectrum. We hypothesized that the lack of consensus in defining PD subtypes can primarily be attributed to two factors: cohort characteristics (varied clinical continuum ranging from de novo to treated cases) and methodological considerations (adjustments for global atrophy and the nature of the input data used in clustering). By employing advanced clustering algorithms and applying them to a large heterogeneous PD multi-cohort dataset, we aimed to overcome previous limitations and establish a comprehensive framework for subtyping PD based on sMRI data.

## Results

### Cohort characteristics

In this study, we included data from four different PD cohorts, each with specific characteristics. The PPMI cohort consisted of PD at early disease stages without medication, while the Barcelona UB-Clínic cohort included medicated patients in relatively more advanced stages. The EXPANd cohort comprised PD in mild-moderate disease stages, and the KIMOVE cohort included a heterogeneous sample of PD. Table 1 provides an overview of the main characteristics for each PD cohort individually, as well as for the entire PD group and the healthy control (HC) group pooled across PPMI, Barcelona UB-Clínic, and EXPANd.

**Table 1:** Cohort descriptives

	PPMI (*N* = 315)	EXPANd (*N* = 75)	KIMOVE (*N* = 136)	Barcelona UB-Clínic (*N* = 107)	PD combined (4 cohorts *N* = 633)	Control group (*N* = 233)
Age, years (median, IQR)	62.37 (14.16)	69 (8)	65.64 (13.56)	65 (16)	64.99 (13.79)	64.5 (13.3)
Sex (men/women)	199/116 (63% men)	44/31 (59% men)	91/45 (67% men)	67/40 (63% men)	401/232 (63% men)	148/85 (64% men)
Disease duration, years (median, IQR)	0.36 (0.53)	4 (6)	2 (5)	6 (9)	1 (3.66)	NA
Medicated (no/yes)	315/0	1/73	58/78	0/107	374/258	NA
Field Strength (1.5 T/3 T)	100/215	0/75	93/43	0/107	193/440	41/192
Education, years (median, IQR)	16 (4)	15 (4)	Missing data	10 (8)	15 (5)	16 (5)
Hoehn & Yahr scale (1/1.5/2/2.5/3/4)	135/0/178/0/2/0	0/0/58/0/17/0	33/0/56/0/19/10	22/5/57/12/10/0	190/5/349/12/48/10	NA
UPDRS part III (median, IQR)	20 (12)	31 (15)	Missing data	13.5 (11)	20 (13)	NA
MoCA (median, IQR)	28 (3)	26 (4)	Missing data	26 (2)	27 (2)	28 (2)

Discrepancies in numerical values are attributed to missing data.

*Barcelona UB-Clínic* Cohort from Hospital Clínic, Barcelona, *EXPANd* cohort from the Exercise in Parkinson’s disease and Neuroplasticity trial, *IQR* interquartile range, *KIMOVE* cohort from the Karolinska Imaging in Movement Disorders study, *MoCA* Montreal Cognitive Assessment, *NA* not applicable, *PD* Parkinson’s disease, *PPMI* cohort from the Parkinson’s Progression Markers Initiative, *UPDRS* Unified Parkinson’s Disease Rating Scale.

### Global atrophy-unadjusted clustering results

**Fig. 1:**
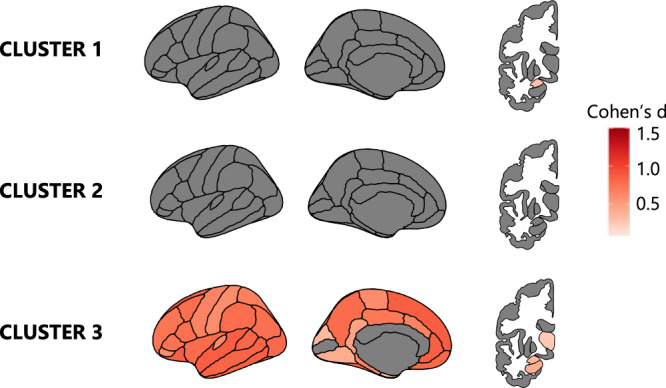
Brain atrophy patterns of the PD clusters without global atrophy adjustment. PD brain atrophy patterns compared to the HC group. The maps represent an average across the right and left hemispheres. Cohen’s d values were calculated and are presented in the figure when the FDR-adjusted *p* value of the ANCOVA reached <0.05. Darker red indicates more atrophy in the PD cluster than in HC. Results were adjusted for age.

When the input of the clustering was not adjusted for global atrophy (severity), the cluster ensemble voting technique identified the 3-cluster solution as the most optimal one in the entire PD sample (Fig. 1), as well as in each individual cohort (Supplementary Fig. 1A), indicating robustness and reliability of the 3-cluster solution. The three clusters showed an increasing gradient of atrophy with significant differences in both total mean cortical thickness (*H(2)* = 499.271, *p* < 0.001) and total subcortical GM volumes (*H(2)* = 55.944, *p* < 0.001) (Supplementary Fig. 2), as well as an increasing white matter hypointensities (WM-hypo) volume (Table 2). Cluster (cl) 3 showed the most pronounced atrophy, higher WM-hypo volume, and yielded the oldest patients with PD (Table 2). Following cl3, cl1 showed intermediate age and subcortical atrophy, while cl2 had no detectable atrophy and yielded the youngest patients with PD. Additionally, cl3 had higher motor impairment, as measured with UPDRS part III, and a higher percentage of individuals with MCI when compared to cl2 (Table 2). There were no differences in disease duration among clusters. Likewise, there were no significant differences in terms of depression, REM sleep behavior disorder (RBD) (Table 2) or the prevalence of Postural Instability and Gait Disorder (PIGD) (χ2 = 0.827, *p* = 0.661), which presented with the following percentages: 27% in cl1, 22% in cl2 and 28% in cl3.

**Table 2:** Demographic and clinical characteristics of the PD clusters not adjusted for global atrophy

	Control group (*N* = 233)	Cl1 (*N* = 302)	Cl2 (*N* = 148)	Cl3 (*N* = 183)	Statistics (H, *p* value)	Post-hoc
Age, years (median, IQR)	64.5 (13.3)	64.40 (14.75)	61.08 (16.11)	67.74 (11.52)	*H* = 40.586 *p* < 0.001	cl1 vs cl2 cl1 vs cl3 cl2 vs cl3
Sex (men/women)	148/85 64% men	190/112 63% men	88/60 59% men	123/60 67% men	χ^2^ = 2.166 *p* = 0.339	
Disease duration, years (median, IQR)	NA	1 (3.65)	1 (2.73)	1.38 (4.63)	*H* = 2.281 *p* = 0.320	
Education, years (median, IQR)	16 (5)	15 (5)	16 (5)	15 (5)	*H* = 2.749 *p* = 0.253	
Hoehn & Yahr scale (1/1.5/2/2.5/3/4)	NA	85/2/174/5/22/6	57/2/73/2/7/2	48/1/102/5/19/2	χ^2^ = 12.013 *p* = 0.284	
Medicated (yes/no) % Medicated	NA	117/184 39%	60/88 40%	81/102 44%	χ^2^ = 1.376 *p* = 0.503	
UPDRS part III (median, IQR)	NA	20 (15)	18 (13)	22 (13)	*H* = 6.309 *p* = 0.043	cl2 vs cl3
MoCA (median, IQR)	28 (2)	27 (3)	28 (3)	27 (3)	*H* = 2.551 *p* = 0.279	
MCI (yes/no) % MCI	14/170 8%	47/184 20%	13/96 12%	38/99 28%	χ^2^ = 9.307 *p* = 0.010	cl2 vs cl3
Depression (presence/absence) % Presence	NA	47/185 20%	26/85 23%	32/110 23%	χ^2^ = 0.536 *p* = 0.765	
RBD (presence/ absence) % Presence	NA	55/99 36%	27/44 38%	37/52 42%	χ^2^ = 0.823 *p* = 0.663	
WM-hypo/eTIV (median, IQR)	0.0007 (0.0006)	0.0010 (0.0010)	0.0008 (0.0006)	0.0015 (0.0014)	*H* = 69.533 *p* < 0.001	cl1 vs cl2 cl1 vs cl3 cl2 vs cl3
Cohort (Barcelona UB-Clínic/EXPANd/KIMOVE/PPMI)	44/38/0/151	46/40/61/155	26/15/36/71	35/20/39/89	χ^2^ = 3.148 *p* = 0.790	

The Kruskal-Wallis test was used for continuous variables and the chi-squared test for categorical variables to compare PD clusters.

*Barcelona UB-Clínic* Cohort from Hospital Clínic, Barcelona, *Cl* cluster, *EXPANd* cohort from the Exercise in Parkinson’s disease and Neuroplasticity trial, *eTIV* estimated total intracranial volume, *IQR* interquartile range, *KIMOVE* cohort from the Karolinska Imaging in Movement Disorders study, *MCI* Mild cognitive impairment, *MoCA* Montreal Cognitive Assessment, *NA* Not applicable, *PPMI* cohort from the Parkinson’s Progression Markers Initiative, *RBD* REM sleep behavior disorder, *UPDRS* Unified Parkinson’s disease rating scale, *WM-hypo* White matter hypointensities.

### Global atrophy-adjusted clustering results

When adjusting the clustering input for global atrophy (severity) as detailed in the methods section, the cluster ensemble voting technique identified the 2-cluster solution as the best cluster solution, followed by the 10-cluster solution. However, the 2-cluster solution yielded large groups (*N* = 472 and *N* = 161) with substantial variability in atrophy patterns within each cluster. Thus, we opted to include the second-best cluster solution for further analysis. From the 10-cluster solution, cl9 and cl10 were excluded due to their small sample sizes of eight and one subject, respectively.

**Fig. 2:**
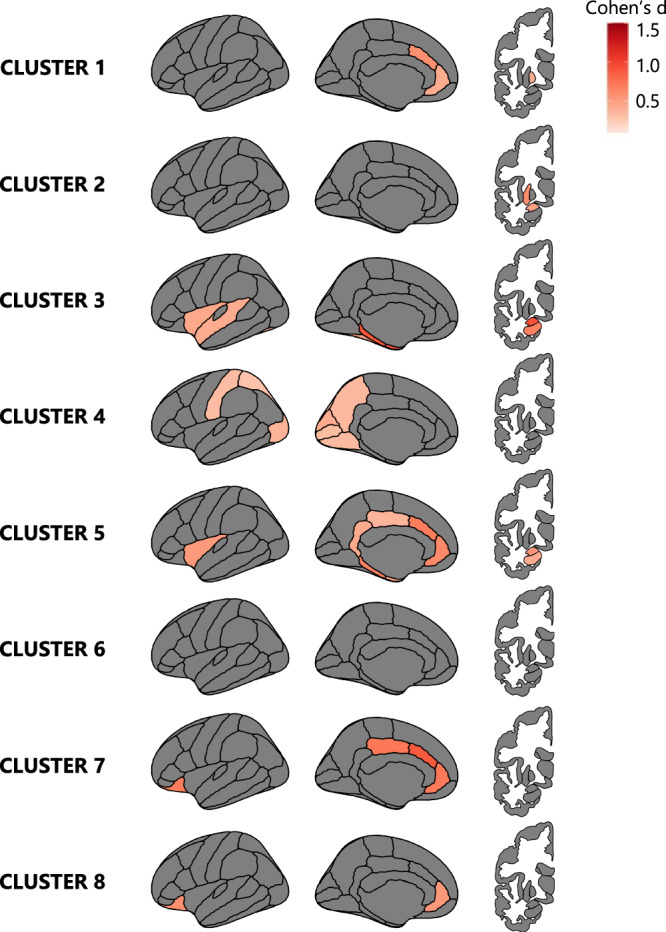
Brain atrophy patterns of the PD clusters adjusted for global atrophy. PD brain atrophy patterns compared to the HC group. The maps represent an average across the right and left hemispheres. Cohen’s d values were calculated and are presented in the figure when the FDR-adjusted *p* value of the ANCOVA reached <0.05. Darker red indicates more atrophy in the PD cluster than in HC. Results were adjusted for age.

The brain patterns of atrophy for the remaining eight clusters are displayed in Fig. 2. In contrast to the global atrophy-unadjusted results (Fig. 1; Supplementary Fig. 1A), when adjusting for severity, the best cluster solution as well as the brain atrophy patterns differed across cohorts (Fig. 2; Supplementary Fig. 1B). Figure 2 shows the atrophy patterns for the eight clusters identified in the entire PD sample (*N* = 633). The eight clusters did not differ in the overall cortical atrophy measured as total mean cortical thickness (*H(7)* = 7.139, *p* = 0.415) (Supplementary Fig. 3). Instead, visual inspection showed distinct atrophy patterns across clusters. Additionally, there were significant differences among clusters in total subcortical GM volume (*H(7)* = 54.943, *p* < 0.001) (Supplementary Fig. 3).

Upon examining the subcortical structures, the clusters could be categorized into those with atrophy in subcortical regions (cl1, cl2, cl3, and cl5) and those without (cl4, cl6, cl7, and cl8). When considering the cortical patterns of atrophy, we could observe four major trends: clusters with atrophy in the anterior cingulate (cl1, cl5, cl7 and cl8); the mediotemporal cortex (cl3) or the parieto-occipital cortex (cl4), and clusters without detectable cortical thinning (cl2 and cl6).

### Clinical characteristics of the PD clusters adjusted for global atrophy

Clusters significantly differed in age, with cl1 and cl8 displaying significantly younger age in comparison to cl2 and cl3. Additionally, cl8 exhibited a younger age distribution than cl4 (Table 3). Clusters did not differ in disease duration. Regarding sex distribution, cl1 exhibited the highest percentage of men, followed by cl7, cl2, and cl5 in descending order. Interestingly, cl4 and cl6 had a higher proportion of women than men. Most of the patients with PD across all clusters were classified in stage 2 according to the H&Y scale (Table 3). Cl1 and cl7 showed lower motor impairment at baseline, as assessed by UPDRS part III, compared to cl4. Furthermore, cl5 displayed slightly better overall cognitive performance, as indicated by higher MoCA scores compared to cl2 (Table 3). It is worth noting that none of the clusters had a median MoCA score below 26, which is typically used as the cutoff to indicate cognitive impairment^13^. Regarding WM-hypo, cl3 showed higher WM-hypo compared to cl1, cl6 and cl8 (Table 3). Despite the lack of significant differences in depression among clusters (χ2 = 9.881, *p* = 0.195), cl4 exhibited the highest prevalence (29%), while cl7 had the lowest (7%) (Table 3). Similarly, there were no significant differences in the frequency of PD with RBD, however there was a trend (χ2 = 12.966, *p* = 0.073) with cl1 (46% RBD), cl2 (44% RBD) and cl4 (45% RBD) showing the highest percentages (Table 3). The prevalence in PIGD did not show significant differences across clusters (χ2 = 7.916, *p* = 0.340). The PIGD cases by cluster were as follows: 34% cl1, 24% cl2, 29% cl3, 31% cl4, 11% cl5, 32% cl6, 17% cl7 and 26% cl8.

**Table 3:** Demographic and clinical characteristics of the PD clusters adjusted for global atrophy

	Control group (*N* = 233)	Cl1 (*N* = 56)	Cl2 (*N* = 189)	Cl3 (*N* = 62)	Cl4 (N = 167)	Cl5 (*N* = 51)	Cl6 (*N* = 46)	Cl7 (*N* = 19)	Cl8 (*N* = 34)	Statistics (H, *p* value)	Post-hoc
Age, years (median, IQR)	64.5 (13.3)	62.15 (14.92)	66.16 (13.90)	68.75 (15.13)	66.56 (12.57)	64.99 (15.26)	62.02 (11.60)	62.00 (16.96)	60.56 (13.00)	*H* = 34.293 *P* < 0.001	cl1 vs cl2 cl1 vs cl3 cl8 vs cl2 cl8 vs cl3 cl8 vs cl4
Sex (men/ women)	148/85 64% men	51/5 91% men	145/44 77% men	40/22 65% men	75/92 45% men	37/14 73% men	19/27 41% men	15/4 79% men	18/16 53% men	χ^2^ = 73.143 *p* < 0.001	cl1 vs cl3 cl1 vs cl4 cl1 vs cl6 cl1 vs cl8 cl2 vs cl4 cl2 vs cl6 cl4 vs cl5
Disease duration, years (median, IQR)	NA	1.74 (6.44)	1 (3.96)	1.73 (4.01)	1.49 (5.62)	0.87 (2.74)	1.13 (2.5)	0.5 (1)	0.83 (1.63)	*H* = 13.470 *p* = 0.061	
Education, years (median, IQR)	16(5)	16 (5)	16 (6)	15 (5)	15 (4)	16 (6)	15 (4)	16 (4)	15 (5)	*H* = 8.857 *p* = 0.263	
Hoehn & Yahr scale (1/1.5/2/2.5/3/4)	NA	19/0/32/1/2/1	58/2/99/2/19/2	12/1/40/2/5/1	45/0/90/6/13/5	15/1/33/0/2/0	14/1/26/0/4/1	11/0/7/0/1/0	14/0/15/1/2/0	χ^2^ = 32.097 *p* = 0.609	
Medicated (yes/no) % Medicated	NA	28/28 50%	79/110 42%	26/36 42%	74/93 44%	17/34 33%	21/25 46%	5/14 26%	7/26 21%	χ^2^ = 11.353 *p* = 0.124	
UPDRS part III (median, IQR)	NA	16 (15)	20 (12)	22 (14)	22 (16)	19 (15)	21 (15)	14.5 (6)	17 (18)	*H* = 22.917 *p* = 0.002	cl1 vs cl4 cl7 vs cl4
MoCA (median, IQR)	28 (2)	28 (2)	27 (3)	26 (4)	27 (3)	28 (2)	27 (3)	28 (3)	28 (3)	*H* = 20.806 *p* = 0.004	cl2 vs cl5
MCI (yes/no % MCI	14/170 8%	8/40 17%	33/105 24%	12/31 28%	23/96 19%	11/33 25%	6/32 16%	2/12 14%	3/21 12.5%	χ^2^ = 5.168 *p* = 0.639	
Depression (presence/ absence) % Presence	NA	7/40 15%	23/114 17%	12/35 26%	36/88 29%	9/35 20%	10/28 26%	1/13 7%	5/20 20%	χ^2^ = 9.881 *p* = 0.195	
RBD (presence/ absence) % Presence	NA	12/14 46%	40/51 44%	10/20 33%	34/41 45%	6/26 19%	5/15 25%	2/10 17%	7/13 35%	χ^2^ = 12.966 *p* = 0.073	
WM-hypo/eTIV (median, IQR)	0.0007 (0.0006)	0.0010 (0.0008)	0.0012 (0.0012)	0.0017 (0.0022)	0.0011 (0.0009)	0.0010 (0.0018)	0.0009 (0.00074)	0.0010 (0.0010)	0.0008 (0.0003)	*H* = 20.049 *p* < 0.001	cl3 vs cl1 cl3 vs cl6 cl3 vs cl8
Cohort (Barcelona UB-Clínic/ EXPANd/ KIMOVE/ PPMI)	44/38/0/151	16/8/6/26	24/25/49/91	13/5/14/30	31/21/39/76	8/5/6/32	10/8/8/20	2/0/5/12	2/3/9/20	χ^2^ = 28.416 *p* = 0.129	

The Kruskal–Wallis test was used for continuous variables and the chi-squared test for categorical variables to compare PD clusters.

*Barcelona UB-Clínic* Cohort from Hospital Clínic, Barcelona, *Cl* cluster, *EXPANd* cohort from the Exercise in Parkinson’s disease and Neuroplasticity cohort, *eTIV* estimated total intracranial volume, *IQR* interquartile range, *KIMOVE* cohort from the Karolinska Imaging in Movement Disorders study, *MCI* Mild cognitive impairment, *MoCA* Montreal Cognitive Assessment, *NA* Not Applicable, *PPMI* cohort from the Parkinson’s Progression Markers Initiative, *RBD* REM sleep behavior disorder, *UPDRS* Unified Parkinson’s disease rating scale, *WM-hypo* White matter hypointensities.

### Clinical progression of the PD clusters adjusted for global atrophy

**Fig. 3:**
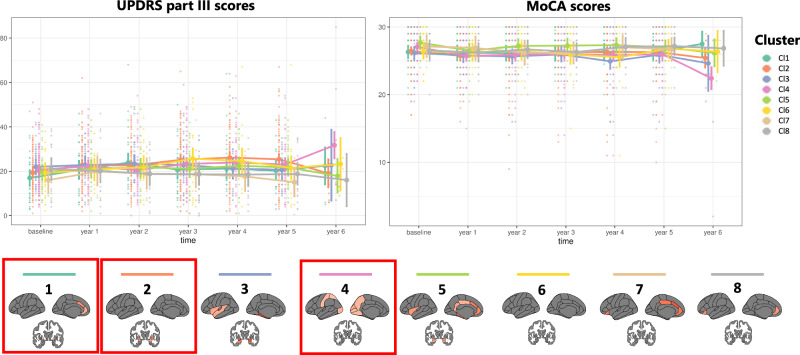
Motor and cognitive trajectories of the PD clusters adjusted for global atrophy over time. Longitudinal analysis of movement impairment and cognitive decline over 6 years follow-up using UPDRS part III and MoCA scores respectively. Background dots represent raw data, with darker dots indicating several individuals with the same score. Foreground lines depict estimated marginal means with error bars based on standard error from the linear mixed effects model. The red squares outline the brain patterns of the clusters showing a significant decline over time (cl1, cl2, cl4 showed significant increase in UPDRS part III scores, while cl4 exhibited significant decline in MoCA scores). Longitudinal UPDRS part III and MoCA scores were available for the Barcelona UB-Clínic and PPMI cohorts. Cl cluster, MoCA Montreal Cognitive Assessment, UPDRS part III Unified Parkinson’s Disease Rating Scale part III.

Global atrophy unadjusted approach resulted in three clusters which were clearly differentiable as they exhibited progressively increasing atrophy, WM-hypo, age, motor, and cognitive characteristics. Therefore, we only characterized the clusters from the global atrophy adjusted approach with longitudinal clinical outcomes available over six years of follow up (Fig. 3). Clusters identified through the global atrophy-adjusted clustering (eight clusters) exhibited significant differences over time in motor scores measured with UPDRS part III (*χ2* = 60.87, *p* = 0.027), with cl1, cl2 and cl4 showing the most pronounced movement impairment over time. Additionally, there was a general trend towards significant differences in cognitive trajectories, measured with MoCA, over time across clusters (*χ2* = 54.89, *p* = 0.072). Post-hoc analyses showed that cl4 had a significant decline in MoCA scores over time, compared with the other clusters.

### Comparison of global atrophy-unadjusted and global atrophy-adjusted approaches

**Fig. 4:**
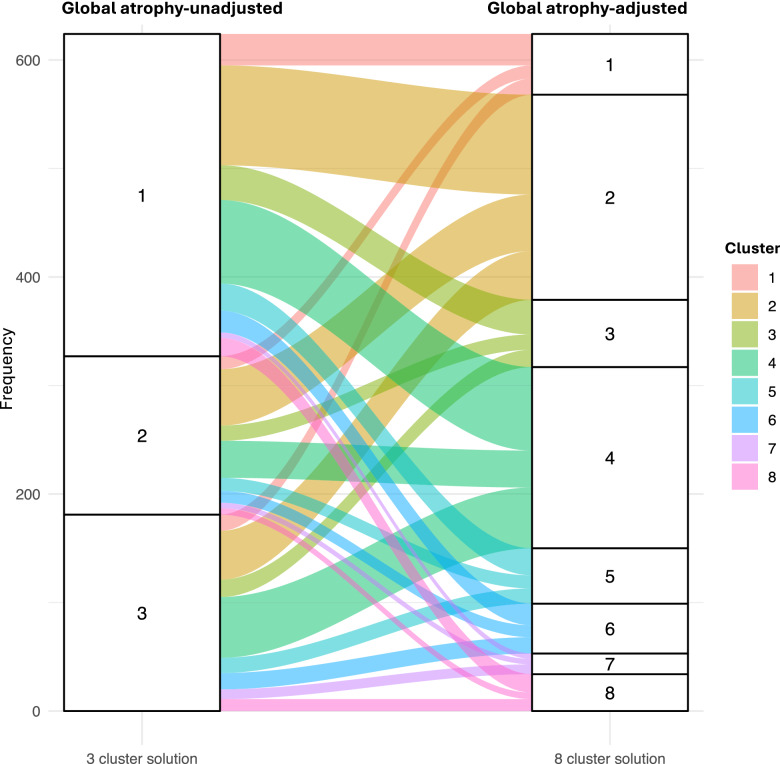
Individual-level cluster transition: global atrophy-unadjusted vs global atrophy-adjusted clustering. Alluvial plot showing the transition of patients with PD between the three clusters obtained from clustering without global atrophy adjustment (left) and the eight clusters obtained from clustering with global atrophy adjustment (right).

The alluvial plot in Fig. 4 reveals the transitions between the 3-cluster solution (not adjusted for global atrophy) and the 8-cluster solution (adjusted for global atrophy), showing a balanced flux of patients with PD between the three and eight clusters (χ2 = 11.11, *p* = 0.677). Examining the dendrograms (Supplementary Fig. 4), the unadjusted cluster analysis shows an inter-cluster distance of up to 0.5 (Supplementary Fig. 4A), indicating notable differentiation among the clusters based on brain atrophy patterns. In contrast, the adjusted version shows a reduced inter-cluster distance of up to 0.25 (Supplementary Fig. 4B), indicating that a large proportion of the variability in the data was explained by severity.

3D multi-dimensional plots (Supplementary Fig. 5) illustrate the unadjusted solution with a U-shaped distribution (Supplementary Fig. 5A), indicating distinctive patterns within the clusters. However, this U-shaped pattern disappears in the adjusted solution, reflecting a more uniform spatial distribution of brain atrophy patterns after accounting for global atrophy (Supplementary Fig. 5B). Given that clustering was conducted in the entire PD sample (Figs. 1 and 2) and validated independently in each individual cohort (Supplementary Fig. 1), we compared the overlap of cluster allocations. Regardless of global atrophy adjustment, we found that clustering allocations in the entire PD sample reasonably overlapped with clustering allocations in individual cohorts (Supplementary Fig. 6), indicating shared brain atrophy patterns.

## Discussion

In our study, we addressed some of the main sources of variability that have contributed to the lack of consensus in defining subtypes in previous literature: the diversity among PD populations, differences in clustering methods used, and the adjustment for global atrophy. We did so by investigating a large PD sample, comprising different disease stages, using cross-sectional clustering based on sMRI. The findings were validated in four different cohorts using the same clustering method. Additionally, we examined the implications of adjusting for global atrophy, used as a proxy of neurodegenerative severity, providing insights into the impact of this adjustment to identify subtypes. We demonstrated (1) the necessity of working with large cohorts encompassing different PD populations to maximize PD heterogeneity; (2) the relevance of employing methodologies that allow to simultaneously consider severity and typicality; and (3) the importance of incorporating both cortical and subcortical regions of interest to ensure an accurate identification and characterization of PD subtypes.

Data-driven subtyping has been employed to provide promising results in the study of subtypes within neurodegenerative disorders^2^. Specifically, structural brain measures have shown to differentiate clinical subtypes^14^ and predict disease progression^15^ in PD, leading to the use of data-driven analyses based on sMRI to identify subtypes. Nevertheless, the lack of a consensus regarding the existence of definitive PD subtypes has persisted, mainly due to methodological and sample differences across studies. Methodologically, sMRI subtyping studies in PD have differed in the way input data for clustering are normalized^6–8^. Depending on whether and how the input data are normalized, the extent to which severity or burden on the brain is captured by the clustering model may differ. Ferreira et al.^12^ proposed a framework to understand AD subtypes based on two dimensions of heterogeneity: typicality and severity. In the current study, we adapted this framework to characterize PD heterogeneity. According to Ferreira et al.^12^ the typicality dimension refers to the extent to which an individual’s clinical and pathological features align with the prototypical presentation of the disease, while the severity dimension (reflected by global atrophy in the current study) focuses on the degree of pathology or neurodegeneration. In our study, *when severity (global atrophy) was not adjusted for*, we observed consistent results across all individual cohorts, as well as in the entire PD sample. In our entire PD sample, we identified three subtypes with increasing degree of atrophy, associated with increasing age and motor impairment. In fact, the topography of atrophy in the subtypes resembled different Braak stages: a subtype without detectable atrophy, followed by a subtype with atrophy in the amygdala (typical of middle Braak stages), and a third one with neocortical atrophy^16,17^. Similarly, Barcelona UB-Clínic and KIMOVE cohorts, which included more advanced PD, exhibited this staging, including a subtype with widespread atrophy in the neocortex. Clustering analyses using clinical data have also identified three distinct subtypes in both de novo PD and more advanced PD characterized by varying degrees of clinical impairment, ranging from a subtype with milder symptoms (“mild” subtype) to a subtype with more pronounced symptomatology (“diffuse malignant”)^4,14^, which had faster clinical progression over time^4^. When examining the patterns of atrophy post-hoc, Fereshtehnejad et al.^14^ found that the “diffuse malignant” exhibited the most substantial atrophy, followed by the intermediate and lastly by the “mild” subtype. Cao et al.^11^ performed a multimodal clustering which incorporated GM volumes and identified two subtypes: the “mild” and the “diffuse malignant”. Furthermore, the absence of significant differences in disease duration but differences in age lend further support to the notion that PD severity correlates with age of onset. Specifically, an older age at onset, as observed in cl3, has been associated with more severe symptoms compared to a younger age of onset^18^.

Regarding the cognitive profiles of our severity-unadjusted subtypes, we did not find differences in global cognitive performance in accordance with previous subtyping literature^6–8^. This finding could be due to the fact that MoCA has limited sensitivity in PD compared to more comprehensive cognitive tests^19^. Furthermore, the cohorts included in the study showed predominantly higher MoCA scores, contributing to a limited range across the MoCA scale and thus maybe failing to adequately represent individuals with lower levels of global cognition. However, when using MCI classification in our study, the subtype with widespread atrophy, older age and highest amount of white matter alterations also presented the highest percentage of PD with MCI (28%), resembling the subtype with extensive GM atrophy described by Inguanzo et al.^8^. Hence, PD with MCI has been described as presenting more GM atrophy and WM alterations than PD without MCI^20^. Similarly, in Dementia with Lewy bodies^21^, in an unadjusted subtyping approach, three sMRI subtypes were described, with one showing more widespread cortical atrophy, older age, the highest amount of WM alterations, and the fastest cognitive decline over time.

In contrast, *when adjusting for global atrophy* *(severity)*, the results did not reflect Braak stages as they did not show the usual progression of pathology from caudal to rostral brain regions. Interestingly, the number of clusters differed across individual cohorts, with the entire PD sample allowing the identification of a higher number of clusters, which suggests that smaller subtypes may be overlooked in smaller cohorts, potentially leading them to be grouped together with other subtypes^22^. Consequently, only when merging all the cohorts we were able to identify the smaller subtypes, which highlights that given the heterogeneous nature of PD, larger samples are needed to capture the full disease spectrum. Clustering based on atrophy measures adjusted for global atrophy, as a measure of severity, has been performed in both de novo^7^ and more advanced PD^6^. In those studies, subtypes did not show a gradient of neurodegeneration but rather distinct atrophy patterns, in accordance with our results. When we adjusted for severity, our results brought out typicality over severity, which allowed us to identify eight PD subtypes in the entire PD sample. The subtypes characterized by a faster increase in motor severity over time were the ones with atrophy in the basal ganglia (cl1 and cl2) or parieto-occipital cortices (cl4). Along these lines, atrophy in the basal ganglia has been associated with motor symptoms in PD^23^. PD subtypes have also been classically divided according to their motor symptomatology into tremor dominant and PIGD subtypes. The PIGD subtype has been associated with a worse course of the disease including a higher frequency of PD with dementia^24^. In addition, RBD has been described as a potential indicator of this subtype^25^ and, interestingly, cl1, cl2 and cl4 exhibited, qualitatively, the highest percentages of RBD. PIGD exhibits more atrophy than tremor dominant in several regions including parietal and occipital regions, and the atrophy described in motor areas has been associated with increased severity in postural instability and gait difficulties^24^. Similarly, and although differences in PIGD prevalence were not significant among subtypes, our subtype with parieto-occipital atrophy (cl4), exhibited the fastest cognitive decline over time. Furthermore, Uribe et al.^7^ described a parieto-occipital subtype characterized by the most severe cognitive impairment. Atrophy in occipital^26,27^ and parietal^27–29^ cortices, as well as hypometabolism in both cortices^30^ has been associated with cognitive impairment in PD. Moreover, parietal, and occipital regions have been described to be the most vulnerable to amyloid-β burden leading to cognitive decline in PD^31^. Another classification that has recently garnered attention is the brain-first versus body-first neurodegeneration pathways in Lewy body disorders. According to this framework, limbic structures would be the earliest to undergo atrophy in brain-first PD^32^. This is consistent with cl1, cl5, cl7 and cl8, which presented with atrophy in the anterior cingulate. Specifically, the olfactory bulb along with the amygdala have been described as the initial site of pathology in brain-first, which aligns with cl5.

Regarding WM burden, WM hyperintensities have been associated to atrophy in the medial temporal lobes^33^. This is consistent with our subtype exhibiting a higher burden of WM-hypo (cl3), which was also characterized by medial temporal atrophy. The global atrophy-adjusted clustering also allowed us to identify subtypes with significant *differences in sex distribution*. Although PD is more frequent in men^34^, by adjusting for severity, we were able to identify subtypes that were more prominent in women with PD than men with PD (cl4 and cl6). None of the two subtypes with a higher frequency of women showed subcortical atrophy. Furthermore, one of these two subtypes was the minimal atrophy subtype, characterized by undetectable atrophy in subcortical and cortical GM regions^6,8^, which was also identified in Albrecht et al.^10^ as the subtype with a higher frequency of women. Accordingly, women with PD have less atrophy in subcortical structures compared to men with PD^35^.

Our global-atrophy adjusted *clustering analyses performed individually in each cohort* showed fewer subtypes in early PD than in more advanced PD, in line with previous literature^6–9^. This finding can be attributed to the fact that atrophy in PD typically emerges after PD onset and increases until later stages together with the development of symptoms^36^. In early PD, previous studies identified two subtypes within the PPMI cohort^7,9^. However, while Uribe et al.^7^ identified two subtypes that differed in their cortical atrophy patterns, the subtypes reported by Wang et al.^9^ showed atrophy only in subcortical structures compared to HC. In contrast to these previous studies, we conducted a comprehensive whole-brain analysis including both cortical and subcortical regions of interest as the input for clustering. In medicated PD, Uribe et al.^6^ identified three subtypes characterized by different patterns of cortical atrophy, including one without detectable atrophy, using vertex-wise analysis. In our study, using a cohort partially overlapping with that in Uribe et al.^6^, (Barcelona UB-Clínic cohort), we identified five subtypes by clustering individuals in the Barcelona-UB Clínic cohort. Similarly to Uribe et al.^6^, we identified two subtypes with different patterns of cortical atrophy but, additionally, instead of identifying only one third subtype characterized by minimal cortical atrophy^6^, we discerned three distinct subtypes with minimal cortical atrophy, each exhibiting varying patterns of subcortical atrophy. These three minimal cortical atrophy subtypes could have been grouped under the same minimal cortical atrophy subtype in Uribe et al.^6^, since they exclusively relied on cortical data. These results emphasize the importance of including both cortical and subcortical brain regions in sMRI subtyping in PD.

Despite the potential of sMRI to track disease progression in PD^36^, there are several major challenges that must be approached to move forward a practical application in clinical studies^37^. Current data-driven PD subtyping approaches lack reproducibility. Additionally, a large disagreement at the individual level across subtyping methods has been highlighted in other neurodegenerative disorders^38^. In our study, we showed that while the unadjusted approach gave evidence of severity, explaining a significant part of brain atrophy differences in PD, it grouped individuals in a broader manner solely based on the extent of the atrophy, without providing information regarding specific patterns of atrophy (typicality) or clinical profiles. On the other hand, adjusting for severity—global atrophy-adjusted results—allowed us to disentangle subtypes, which were characterized by different clinical and demographic profiles. This fact was emphasized by our alluvial plot, which showed that the eight clusters (typicality) were represented in each of the three severity clusters (severity). Moving beyond a cross-sectional design, longitudinal subtyping approaches may be more effective in disentangling the dimensions of typicality and severity by tracking atrophy trajectories. However, trying to describe the progression of cross-sectional PD subtypes on post-hoc analyses is challenging, especially in less frequent PD subtypes with high rates of attrition^39^. Going one step further, Zhou et al.^40^ used the Subtype and Stage Inference (SustaIn) algorithm applied to clinical data and imaging data from five regions of interest and identified two PD subtypes characterized by different patterns of disease progression. Additionally, the subtype with worst prognosis exhibited a poor levodopa response. Beyond cross-sectional data, longitudinal clustering based on sMRI longitudinal measures has been developed and successfully applied in AD^41^ and healthy ageing^42^, allowing to track progression of atrophy patterns which can help predict differential courses of the disease. We believe that applying subtyping based on longitudinal sMRI in PD will help to better characterize the disease at a more individualized level.

While this study offers valuable insights into PD subtyping, there are some limitations that need to be considered. Firstly, we focused solely on sMRI. While it enables the investigation of neurodegeneration, combining multiple imaging modalities could provide a better comprehension of subtypes. Additionally, averaging left and right brain regions to reduce the number of features entered in the analysis may have hidden potential asymmetry in the atrophy patterns. Secondly, the cross-sectional nature of this study restricts our ability to explore the progression of identified subtypes. To partially address this limitation, we tracked cognitive and motor data longitudinally for cohorts where such information was available. However, the absence of longitudinal data across all cohorts limited our capacity to fully elucidate the trajectories of the subtypes. Additionally, the medication status could have influenced the longitudinal motor outcomes, particularly when using *on* state motor scores in a sample comprising both unmedicated and medicated individuals, including those transitioning from unmedicated de novo PD to medicated PD over time.

In conclusion, our study elucidates the factors contributing to the inconsistency observed in reported atrophy subtypes across previous PD studies, by emphasizing two crucial considerations for future research. First, to identify robust subtypes, the target PD population needs to be representative and inclusive of a broader clinical continuum in PD. Second, it is crucial to establish a balance between the dimensions of severity and typicality, to comprehensively associate different patterns of brain atrophy with clinical profiles in PD.

## Methods

### Participants

This study incorporated data from four different cohorts, comprising a total of 633 individuals with PD: (1) the Parkinson’s Progression Markers Initiative (*PPMI*) encompassing 315 unmedicated de novo PD; (2) patients from the Parkinson’s disease and Movement Disorders Unit at Hospital Clínic and the University of Barcelona in Barcelona, Spain (*Barcelona UB-Clínic*), comprising 107 PD who were undergoing medication; (3) the Karolinska Imaging in Movement Disorders Study (*KIMOVE*) conducted at Karolinska University Hospital (Sweden), involving 136 PD; and (4) the Exercise in Parkinson’s disease and Neuroplasticity (*EXPANd*) study, also conducted at Karolinska University Hospital (Sweden), encompassing 75 participants with PD. PPMI data used in the preparation of this article were obtained [April, 30, 2023] from the PPMI database (www.ppmi-info.org/access-data-specimens/download-data), RRID:SCR_006431 upon request after approval by the PPMI Data Access Committee. For up-to-date information on the study, visit www.ppmi-info.org. In the PPMI cohort, patients with PD who were taking antiparkinsonian medication were excluded; while in the Barcelona UB-Clínic cohort, only patients with PD taking antiparkinsonian drugs were included. In addition, PPMI and Barcelona UB-Clínic cohorts had clinical data for up to six years of follow-up. EXPANd and KIMOVE cohorts did not have any specific requirements regarding the medication status for inclusion in the study. The KIMOVE cohort consisted of patients with PD from the Karolinska University Hospital Neurology outpatient clinic who had underwent MRI examination at the Karolinska University Hospital Radiology Department as part of their clinical workup. This cohort included individuals both with and without medication. In EXPANd, participants had mild-moderate stage PD, and were recruited in a context where individuals were chosen for an intervention. Consequently, they needed to be suitable candidates, even though in the current study the sample was pre-intervention. Therefore, the inclusion criteria required absence of other disorders significantly impacting balance, voice, or speech performance; as well as not experiencing severe states of tremor, dyskinesia, or dystonia, nor present with inability to hear instructions without hearing aid^43^. PD with a diagnosis of dementia were excluded in all the cohorts. Additionally, analyses included data from 233 HC, including 151 from PPMI, 44 from Barcelona UB-Clínic and 38 from EXPANd. Participants with sMRI scans exhibiting motion artifacts, poor image quality or failed FreeSurfer segmentation were excluded. Local ethics committee at each center approved the study. All participating PPMI sites received approval from an ethical standards committee prior to study initiation, for a list of participant sites see https://www.ppmi-info.org/about-ppmi/ppmi-clinical-sites. Barcelona-UB Clínic dataset was collected under a protocol approved by the University of Barcelona and Hospital Clínic bioethical committees: Institutional Review Board (IRB00003099). KIMOVE was approved by the Regional Ethical Review Board in Stockholm 2015/1607-31. EXPANd was approved by the Regional Ethical Review Board in Stockholm 2016/1264–31/4, 2017/125832 and 2017/2445–32. Written consent on participation was obtained from all patients or appropriate surrogates according to the Declaration of Helsinki, except for the KIMOVE cohort where informed consent was waived due to the retrospective nature of the study.

### Clinical assessments

Treatment with antiparkinsonian drugs consisted of different combinations of L-dopa, catechol-O-methyltransferase inhibitors, monoamine oxidase inhibitors, dopamine agonists, and amantadine. Motor symptoms were assessed using part III of the Unified Parkinson’s Disease Rating Scale (UPDRS^44^) and the Movement Disorder Society-Sponsored Revision of the Unified Parkinson’s Disease Rating Scale (MDS-UPDRS^45^). Disease staging was assessed with either the original or the modified version of the Hoehn and Yahr scale (H&Y). Global cognitive performance was assessed using the Montreal Cognitive Assessment scale (MoCA^13^) in PPMI and EXPANd, and the Mini-Mental State Examination (MMSE^46^) in Barcelona UB-Clínic. To ensure comparability across cohorts, we converted the MMSE scores to MoCA scores using a previously established conversion method^47^. The presence of mild cognitive impairment (MCI) was determined in the Barcelona UB-Clínic and EXPANd cohorts using the Litvan criteria II, and in the PPMI cohort using the Litvan criteria I^48^. Participants undergoing medication were in the *on* state during assessments. Depression was evaluated using standardized scales, with higher values than the specified cut-offs indicating its presence: the Geriatric Depression Scale^49^ in PPMI using a cut-off of 5, the Hospital Anxiety and Depression Scale^50^ in EXPANd with a cut-off of 8, and the Beck Depression Inventory^51^ in Barcelona UB-Clínic with a cut-off of 10. The REM Sleep Behavior Disorder Questionnaire with a cut-off of 5 was available for the PPMI cohort to determine presence of RBD. Presence or absence of PIGD was assessed in individuals within PPMI and EXPANd cohorts.

### sMRI data

3D T1-weighted images were acquired from all participants using either 1.5 or 3 Tesla scanners. Images were managed through the hive database system (theHiveDB)^52^, and preprocessed at Karolinska Institutet. Atrophy measures of cortical and subcortical regions were obtained using FreeSurfer version 7.1, and subsequently averaged across left and right hemispheres, resulting in 34 cortical (Desikan-Killiany atlas) regions of interest (ROIs) measured by cortical thickness, and seven subcortical ROIs measured by gray matter (GM) volumes. To assess white matter alterations, WM-hypo were obtained using FreeSurfer 7.1. GM volumes and WM-hypo were adjusted for the estimated total intracranial volume (eTIV) with the residual method with HC as the reference group^53^.

### sMRI clusters based on data-driven analysis

When studying sMRI subtyping in neurodegenerative disorders, it is important to consider whether subtypes with different brain atrophy patterns may be confounded by the global burden of the disease on the brain^22^. This confounder reflects *severity*, represented as an sMRI-based biological entity (e.g., global atrophy) capturing the overall disease progression and burden in the brain^12^. However, the impact of adjusting (or lack thereof) for global atrophy on identification of subtypes in PD has not been tested in previous studies. Thus, in this study, we compared the global atrophy-unadjusted and global atrophy-adjusted approaches in clustering-based subtyping. Analyses were conducted using R version 3.6.3.

### Global atrophy-unadjusted clustering

Prior to clustering we adjusted the sMRI-atrophy measures (thickness and volume measures) for potential confounding variables. Thickness and volume measures in the PD and HC groups were adjusted for field strength (1.5 T vs 3.0 T) using a residual approach^53^. GM volumes of subcortical structures and the hippocampus were additionally adjusted for eTIV. The HC group (combined across EXPANd, PPMI, Barcelona UB-Clínic) was used as the reference group. Further, we controlled for the systematic differences observed in thickness and volume measures when combining data across multiple cohorts using a residual approach^41,53^ and taking the PPMI cohort as the reference group due to its largest sample size. Then, we performed a cluster analysis on the PD sample with the random forest method^54^ applied to the 41 adjusted sMRI measures. The random forest method provided a similarity matrix followed by dimension reduction with multidimensional scaling. Lower dimensional representation from multidimensional scaling was used as the input data for the hierarchical clustering using the average linkage method^55^. The effectiveness of this approach in delivering robust and accurate clustering results has been demonstrated in previous studies^21,56^. We performed the data-driven analysis on the entire PD sample, which included all cohorts (*N* = 633), and validated the results in each cohort individually.

### Global atrophy-adjusted clustering

We also performed the cluster analysis after adjusting the sMRI-atrophy measures for global atrophy as a measure of biological severity. For the global atrophy adjustment, cortical thickness measures were adjusted by total mean cortical thickness, and GM volumes were adjusted by eTIV-adjusted total GM volume at the individual level^6,57^. sMRI-atrophy measures adjusted for field strength, cohort, global atrophy, and additionally for eTIV in the case of subcortical/volumetric ROIs, were used as the input data for the hierarchical clustering using the average linkage method. The remainder of the clustering steps were identical to the model described in the global atrophy-unadjusted model above. We performed the data-driven analysis on the entire PD sample, which included all cohorts (*N* = 633), and additionally validated the results in each cohort separately.

### Assessment of clustering models

We used a cluster ensemble voting technique to identify the optimal cluster solution. This method involved considering the entire decisions of 26 indices, which were part of the NbClust package in R^58^ which included the Calinski-Harabasz and Dunn indices. Additionally, we employed visual inspection of the dendrogram (Supplementary Fig. 4). To ensure statistical power for post-hoc characterization of the clusters and generalization of the results, the sample size of each cluster was considered, excluding clusters with fewer than 10 subjects.

Multidimensional scaling of input to clustering (random forest-based proximity of individuals based on the 41 ROIs) was used to represent the data as three components (dimensions). We chose three components to be the optimal low-dimensional representation of the input data using scree plots. These three components were visualized with multidimensional plots illustrating the distribution of clusters. Furthermore, we created an alluvial plot to visualize how individuals with PD were assigned to different clusters in each approach and to identify any discrepancies or consistencies between the clustering methods.

### Characterization of the clusters

Subsequent analyses were performed to characterize PD clusters based on demographic and clinical variables. Additionally, we compared global atrophy measures—the total mean cortical thickness and the eTIV-adjusted total subcortical GM volume—across clusters (both measures adjusted for field strength and cohort). Clusters were also assessed in terms of their WM-hypo burden. The Kruskal-Wallis test was used to analyse continuous variables, while the chi-square test was employed for categorical variables. Tests were two-sided. To further characterize the clusters, we analysed the longitudinal trajectories of motor and cognitive outcomes (assessed by UPDRS part III and MoCA, respectively) from baseline to follow-up up to a 6-year period in PPMI and Barcelona UB-Clínic (Supplementary Table 1). We modeled longitudinal change in motor and cognitive scores using linear mixed effects models including random intercept for each subject. Motor and cognitive scores were treated as the dependent variable while the interaction of time, cluster allocation and age were treated as independent variables. Linear mixed effects models were controlled for multiple comparisons using the Tukey adjustment in post-hoc analyses.

ANCOVA corrected for multiple comparisons with false discovery rate (FDR), were employed to compare the sMRI atrophy measures averaged between the left and right hemispheres of each PD cluster with the HC group (*N* = 233). In ANCOVA, sMRI atrophy measures were adjusted for field strength and cohort, as well as eTIV in the case of subcortical GM volumes. Cohen’s d values were calculated for age-adjusted thickness and volume measures to visually compare each cluster with HC when the *p* value of the ANCOVA reached significance. The ggseg package from R^59^ was used to display the brain maps, enabling the comparison of the brain atrophy patterns between the PD clusters and the HC group, which was used as the reference.

### Supplementary information

Supplementary materials

## Data Availability

Parkinson’s Progression Markers Initiative (PPMI) cohort is a publicly available datasets that can be found at: http://www.ppmi-info.org. Data from EXPANd, KIMOVE and Hospital Clínic de Barcelona cohorts are not publicly available (due to EU legislation); however, upon reasonable request, data sharing is possible and will be regulated through a data transfer and user agreement.
