# Supplementary material for: MRI subtypes in Parkinson’s disease across diverse populations and clustering approaches
Source: NPJ Parkinsons Dis. 2024 Aug 16;10:159. doi: 10.1038/s41531-024-00759-2 (PMC11329719; doi:10.1038/s41531-024-00759-2)
Supplement: Supplementary file 1 — Supplementary materials [file 41531_2024_759_MOESM1_ESM.pdf]

Supplementary Figure 1. Brain atrophy patterns of each cohort individually.

(A) Global atrophy-unadjusted

PPMI cohort | **EXPANd cohort** | **KIMOVE cohort** | **Barcelona UB-Clínic cohort**

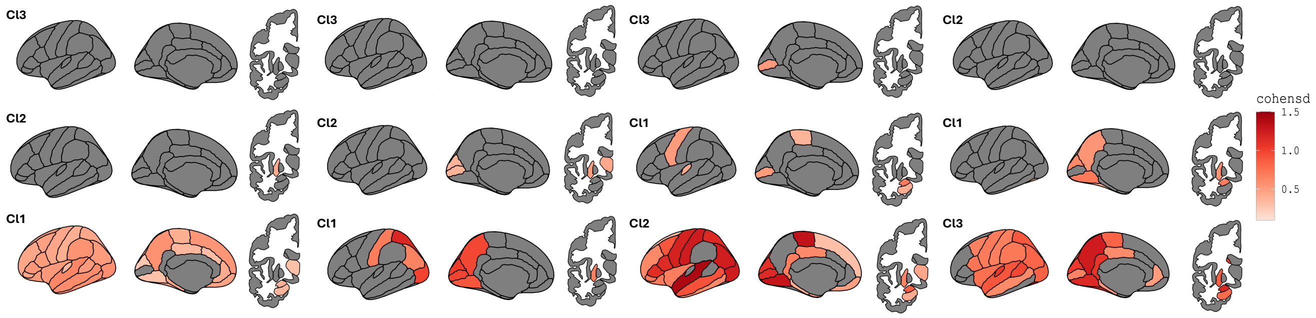

PPMI cohort comprised **newly diagnosed** PD individuals that were not taking antiparkinsonian medication at the moment of assessment.

EXPANd cohort participants had **mild-moderate stage** PD. Inclusion criteria required not experiencing severe states of tremor, dyskinesia, or dystonia.

KIMOVE cohort included individuals both with and without antiparkinsonian medication who underwent an MRI scan as part of their **clinical workup**.

Barcelona UB-Clínic cohort consisted of PD individuals in relatively more **advanced disease stages** taking antiparkinsonian **medication**.

(B) Global atrophy-adjusted

PPMI cohort | **EXPANd cohort** | **KIMOVE cohort** | **Barcelona UB-Clínic cohort**

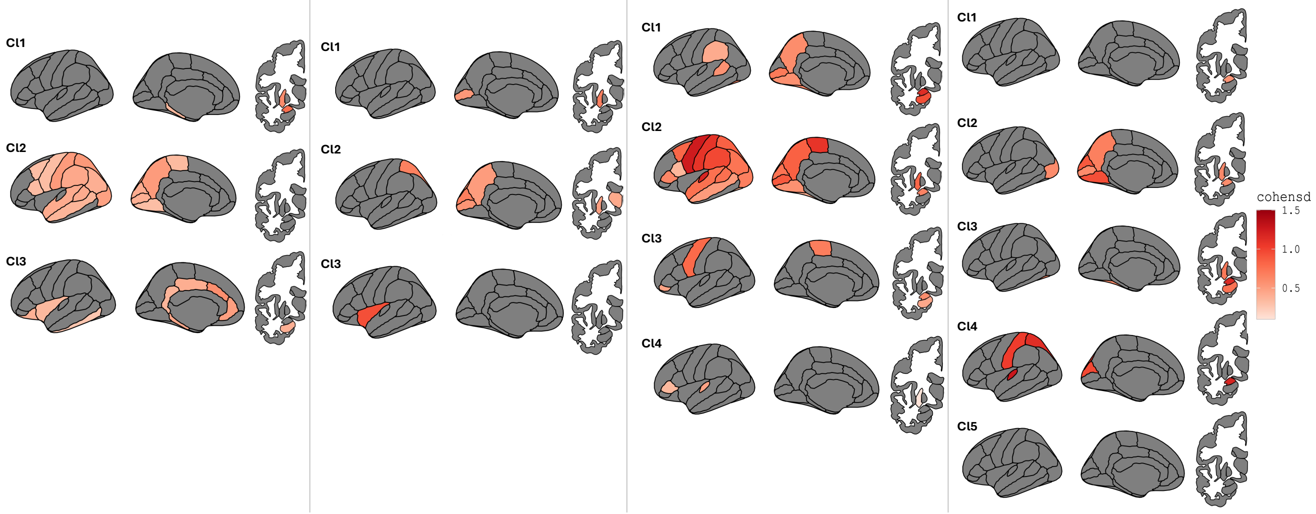

PPMI cohort comprised **newly diagnosed** PD individuals that were not taking antiparkinsonian medication at the moment of assessment.

EXPANd cohort participants had **mild-moderate stage** PD. Inclusion criteria required not experiencing severe states of tremor, dyskinesia, or dystonia.

KIMOVE cohort included individuals both with and without antiparkinsonian medication who underwent an MRI scan as part of their **clinical workup**.

Barcelona UB-Clínic cohort consisted of PD individuals in relatively more **advanced disease stages** taking antiparkinsonian **medication**.

Parkinson's disease brain atrophy patterns compared to the healthy control group. The maps represent an average across the right and left hemispheres. Cohen's d values were calculated and are presented in the figure when the p-value of the ANCOVA reached <0.05. Darker red indicates greater atrophy in Parkinson's disease clusters compared to the healthy control group. Results were adjusted for age.

Supplementary Figure 2. Overall atrophy measures across the global atrophy-unadjusted clusters identified in the entire PD sample.

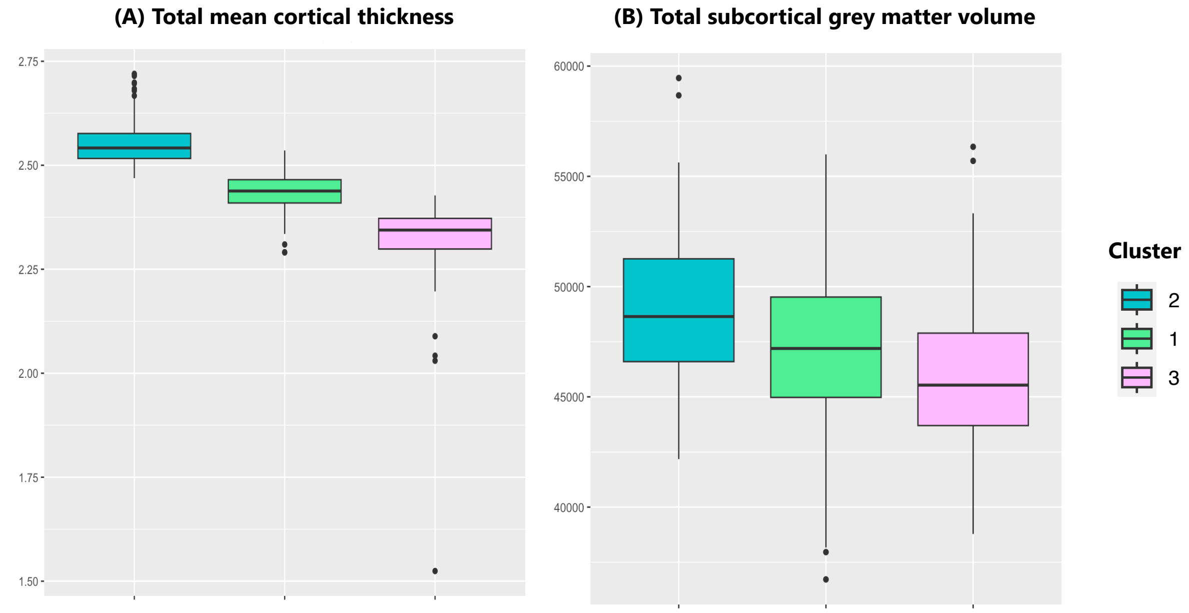

Boxplots displaying (A) total mean cortical thickness and (B) total subcortical grey matter volumes across the three PD clusters obtained from global atrophy-unadjusted clustering. All pairwise comparisons were significant ($p<0.05$). Abbreviations: CTh – cortical thickness; GM – grey matter.

Supplementary Figure 3. Overall atrophy measures across the global atrophy-adjusted clusters identified in the entire PD sample.

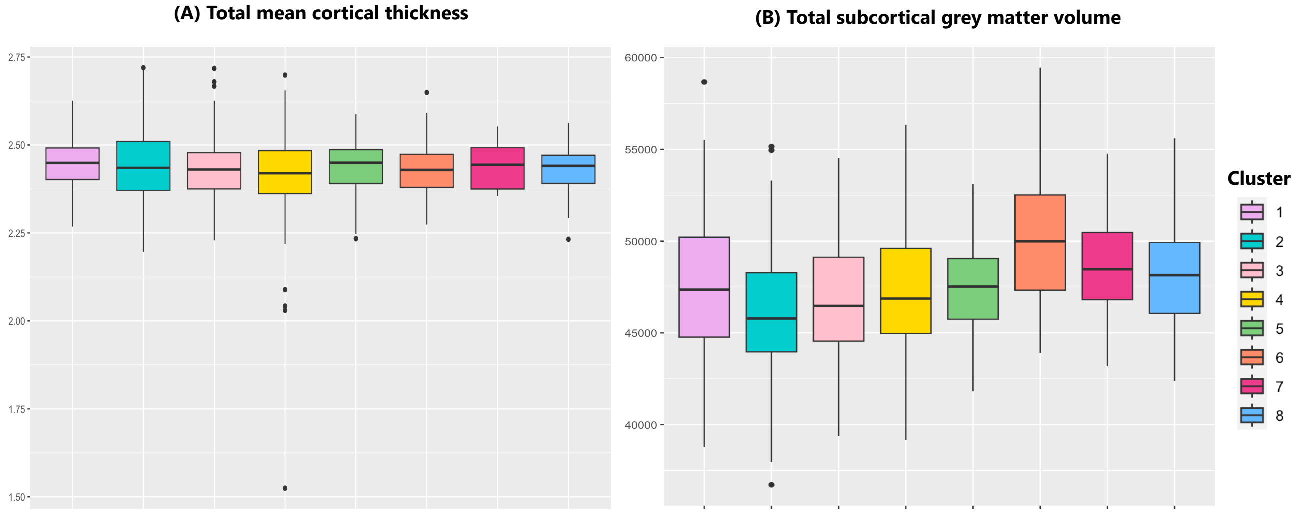

Boxplots displaying (A) total mean cortical thickness and (B) total subcortical grey matter volumes across the eight PD clusters obtained from global atrophy-adjusted clustering. PD subtypes did not show significant differences in total mean cortical thickness (A). However, significant differences ($p<0.05$) in total subcortical grey matter (B) were observed between the following pairwise comparisons: cl2 vs cl4; cl2 vs cl6; cl2 vs cl8; cl6 vs cl1; cl6 vs cl3; cl6 vs cl4; cl6 vs cl5. Abbreviations: cl - cluster; CTh – cortical thickness; GM – grey matter.

Supplementary Figure 4. Dendrograms from the cluster analyses.

(A) Global atrophy-unadjusted **(B) Global atrophy-adjusted**

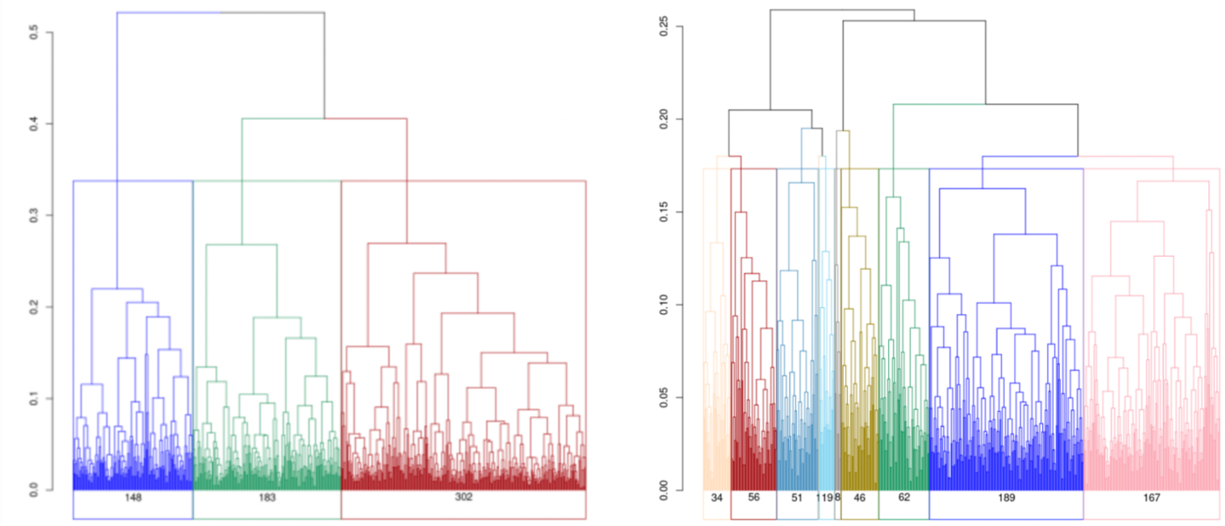

Dendrogram or tree structure split and colour-coded based on the optimal cluster solution for each clustering model. (A) The 3-cluster solution is shown for clustering without adjustment for global atrophy, and (B) the 10-cluster solution is shown for clustering with adjustment for global atrophy. The vertical axis shows the inter-cluster distance based on the random forest similarity between individuals.

Supplementary Figure 5. Multidimensional scaling.

(A) Global atrophy-unadjusted **(B) Global atrophy-adjusted**

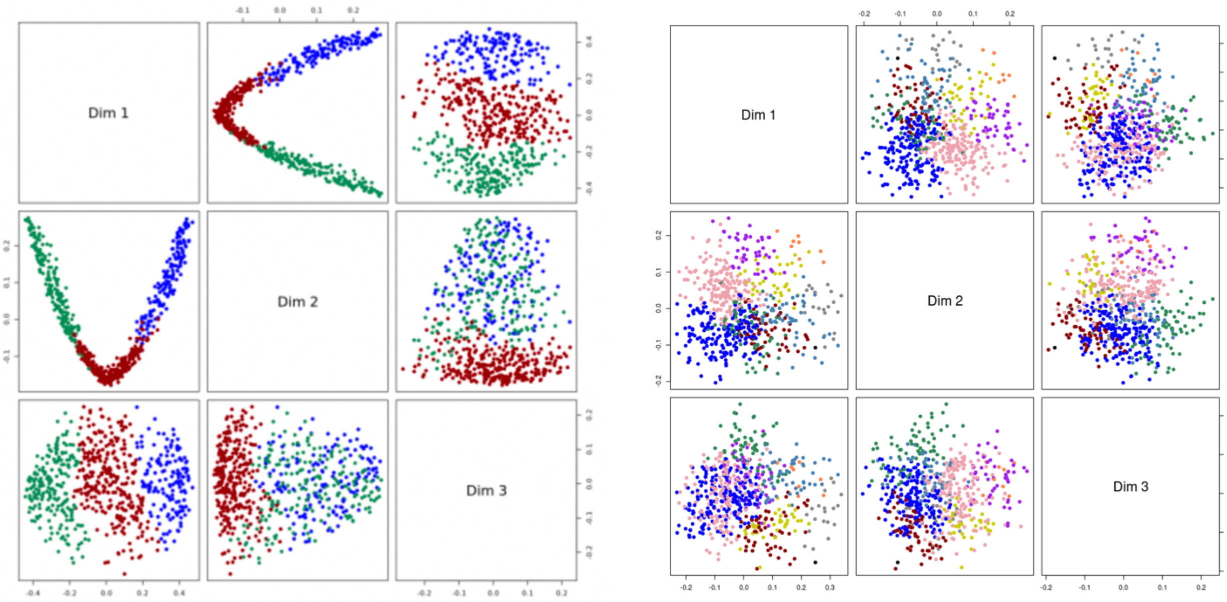

3D multi-dimensional scaling representation of the similarity matrix. The plots represent the optimal cluster solution for each clustering model. (A) The 3-cluster solution is shown for clustering without adjustment for global atrophy, and (B) the 10-cluster solution is shown for clustering with adjustment for global atrophy. Dots represent PD subjects. The distance between them symbolizes how similar the subjects are with respect to their patterns of brain atrophy, and the colour denotes the clusters obtained by the hierarchical clustering.

Supplementary Figure 6. Clustering allocation comparison between the entire PD sample clustering and individual cohort clustering.

(A) Global atrophy-unadjusted

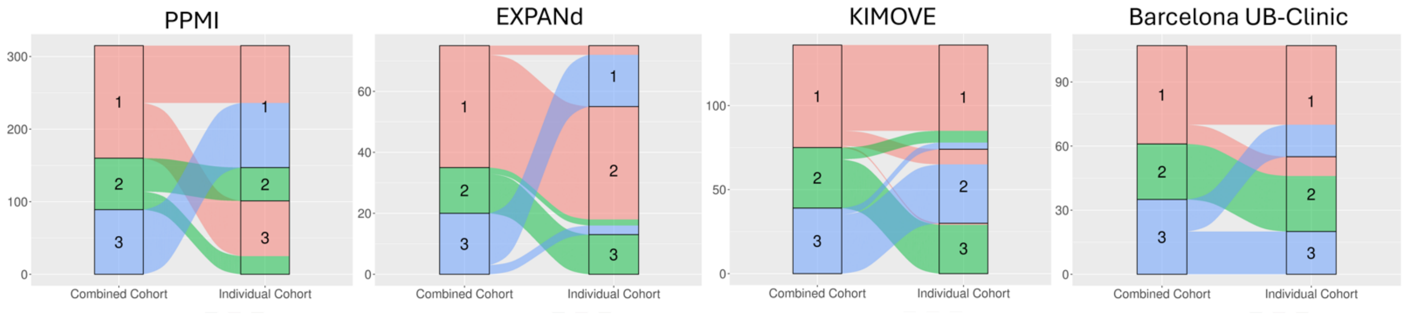

(B) Global atrophy-adjusted

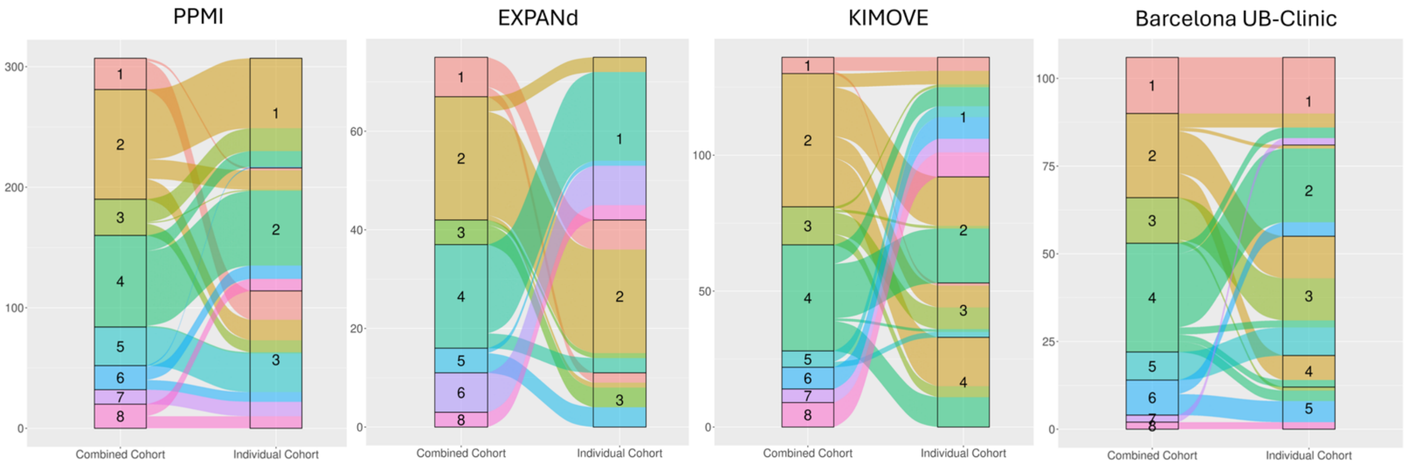

Supplementary Table 1. Breakdown of samples contributing to clinical progression models for UPDRS part III and MoCA.

	Baseline visit	1-year follow up	2-year follow up	3-year follow up	4-year follow up	5-year follow up	6-year follow up
**UPDRS-III**							
**PPMI**	310	273	280	272	244	217	12
**Barcelona UB-Clínic**	104	6	3	61	0	40	17
**MoCA**							
**PPMI**	310	273	280	272	244	217	12
**Barcelona UB-Clínic**	104	9	0	0	0	45	22

Abbreviations: Barcelona UB-Clínic – Cohort from Hospital Clínic, Barcelona; MoCA – Montreal Cognitive Assessment; PPMI – cohort from the Parkinson's Progression Markers Initiative; UPDRS – Unified Parkinson's Disease Rating Scale.
